# Reply of the authors: Positioning human chorionic gonadotropin therapy for androgen users within a harm reduction framework

**DOI:** 10.1016/j.xfre.2025.08.002

**Published:** 2025-12-11

**Authors:** Diederik L. Smit, Tijs Verdegaal, Peter Bond

**Affiliations:** a Department of Performance and Image-enhancing Drugs Research, Android Health Clinic, Utrecht, the Netherlands; b Department of Internal Medicine, Zaans Medical Centre, Zaandam, the Netherlands; c Spaarne Gasthuis Academy, Spaarne Gasthuis, Haarlem, the Netherlands

We thank Dokania et al. (1) for their thoughtful response and careful reading of our study on human chorionic gonadotropin (hCG) therapy in androgen users with impaired spermatogenesis (2). We appreciate their recognition of the real-world challenges faced by this population and agree that it deserves further clinical and scientific attention.

The points raised—such as the absence of a control group, omission of sperm morphology, and lack of endocrine biomarkers—are valid and align with limitations we acknowledged. Rather than reiterating these, we wish to emphasize the clinical relevance of engaging with this underserved group and outline directions for future research.

Ultimately, the aim should be to avoid intrauterine insemination or in vitro fertilization treatment, which often places a substantial physical and emotional burden on the female partner because of, among other things, the required hormonal therapy (3), by restoring spermatogenesis through noninvasive, low-risk strategies. Future studies should refine hCG protocols, exploring optimal dose and duration, its effectiveness during continued testosterone exposure, the impact of androgen type and dose, and the potential additive value of recombinant follicle-stimulating hormone. Time to conception should be included as a clinically meaningful endpoint. In addition to standard semen parameters—including morphology—assessing sperm deoxyribonucleic acid fragmentation may be particularly informative because the impact of anabolic androgen use on deoxyribonucleic acid integrity remains unclear and underexplored.

It is also essential to evaluate the role of this therapy within a harm reduction framework (4). Key questions include whether medically supervised hCG alongside a maintenance dose of testosterone leads to more moderate use and how adherence compares with other strategies: no treatment, cessation with expectant recovery, or hCG initiation after cessation. For the latter, efficacy is well established, and it often maintains testosterone levels to a sufficient degree to prevent hypogonadal symptoms (5). We hypothesize that proactive engagement fosters trust and therapeutic alliance, which may in turn create opportunities to reduce or discontinue androgen use once fertility goals are met. In contrast, refusing support or demanding abstinence may unintentionally reinforce unsupervised or escalating use.

We believe that continued investigation and pragmatic engagement are vital to advancing evidence-based, patient-centered fertility care. Insights from this group may also inform strategies for men receiving therapeutic testosterone who wish to preserve fertility.

## CRediT Authorship Contribution Statement

**Diederik L. Smit:** Writing – original draft. **Tijs Verdegaal:** Writing – review & editing. **Peter Bond:** Writing – review & editing.

## Declaration of Interests

D.L.S. has nothing to disclose. T.V. has nothing to disclose. P.B. has nothing to disclose.
